# PyComplexHeatmap: A Python package to visualize multimodal genomics data

**DOI:** 10.1002/imt2.115

**Published:** 2023-05-25

**Authors:** Wubin Ding, David Goldberg, Wanding Zhou

**Affiliations:** ^1^ Center for Computational and Genomic Medicine The Children's Hospital of Philadelphia Philadelphia Pennsylvania USA; ^2^ Department of Pathology and Laboratory Medicine University of Pennsylvania Philadelphia Pennsylvania USA

## Abstract

PyComplexHeatmap was designed to visualize matrix data and associated metadata through sophisticated, richly annotated heatmap layouts. We have integrated the R grammar‐of‐graphics semantics with the Python‐native matplotlib/Pandas‐based data science ecosystem, allowing users to utilize built‐in matplotlib colormaps and project Pandas DataFrame directly as horizontal or vertical color bars. PyComplexHeatmap supports high‐level data abstraction and can display up to five data dimensions within a single heatmap visualization.

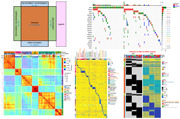

## INTRODUCTION

Advancements in science and technology in the field of biology, including high‐throughput sequencing, have led to an increase in sample size and sequencing coverage. To handle the ever‐growing data volume, numerous Python software and algorithms have been developed. For example, SCANPY [1], EpiScanpy [2], and scVelo [3] are useful for processing and analyzing single‐cell sequencing data, while PyDESeq2 [4] and GSEApy [5] facilitate differential expression gene analysis and gene set enrichment analysis, respectively. Squidpy [6] is a recently introduced package for spatial single‐cell analysis. Furthermore, Python is widely employed for machine learning and deep learning. An increasing number of Python packages are being developed to address specific challenges, such as imputing missing values in single‐cell sequencing data [7] and integrating single‐cell omics data [8] using deep learning.

Despite the popularity of Python in machine learning, deep learning, and big data processing, there is currently no powerful package for generating complex heatmaps similar in caliber to R's *ComplexHeatmap* [9, 10]. This has become a pressing issue for researchers who utilize Python for data analysis, machine learning, and deep learning. Consequently, there is an urgent need for a Python package capable of generating highly complex heatmaps.

To fill this gap in Python, we developed *PyComplexHeatmap*, a Python package that enables users to easily visualize multidimensional biological data. We have added several new features to enhance user‐friendliness while accommodating the Python environment. *PyComplexHeatmap* possesses multiple features that are not included in the current state‐of‐the‐art Python heatmap library *Seaborn* [11]. With *PyComplexHeatmap*, users can: (i) Plot various types of rows or columns annotations (e.g., simple heatmap annotation, barplot annotation, boxplot annotation, scatterplot annotation, and label annotations) separately or together with the main heatmap, with the ability to add group labels on the top of simple heatmap annotations by simply turning on a parameter. (ii) Add label annotations for specific rows or columns quickly and evenly distribute the labels throughout the axis without extra manual editing. (iii) Easily choose different palettes using python matplotlib [12] build‐in colormap, custom colormap, or colors mapping dict. (iv) Combine two or more heatmaps horizontally or vertically to create a joint figure. (v) Visualize up to five variables in the data using a dot heatmap.

## RESULT

### Implementation

**Figure 1:**
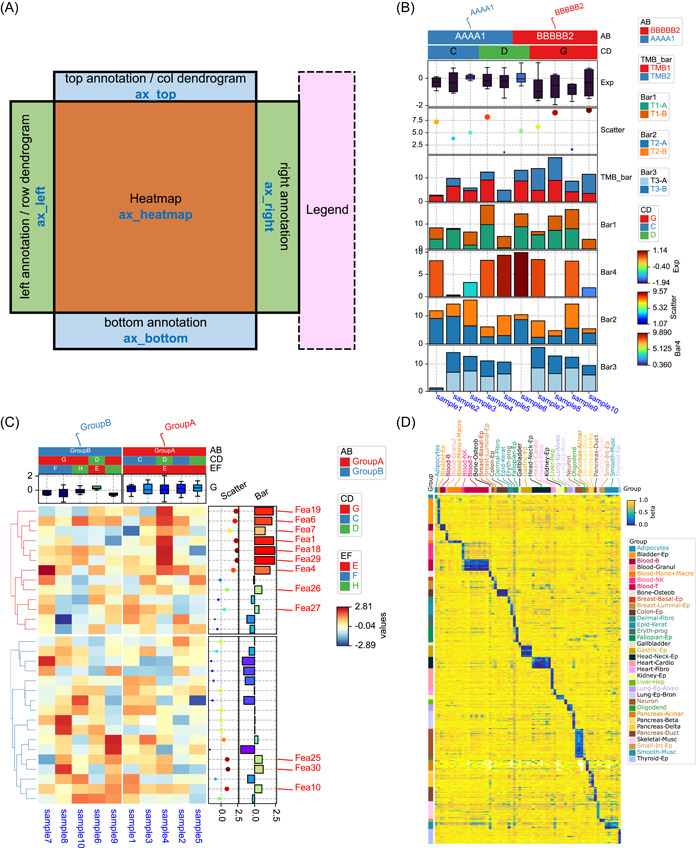
Layout and example heatmaps generated by PyComplexHeatmap. (A) The layout of PyComplexHeatmap. (B) Heatmap annotation is plotted separately to visualize the simulated categorical and continuous data simultaneously. (C) Heatmap annotation is plotted alongside the main heatmap. In addition to rows and columns annotations, a label annotation is included to emphasize the interested rows. (D) Heatmap of cell type‐specific CpG methylation markers in different groups from the Loyfer2023 data set.

*PyComplexHeatmap* is a Python package that is built on a few essential Python libraries, including matplotlib [12], pandas (https://github.com/pandas-dev/pandas), scipy [13], and numpy [14]. As illustrated in Figure 1A, the layout of PyComplexHeatmap includes the column annotations (top and bottom), row annotations (left and right), main heatmap (optionally split up into multiple rows and columns), and figure legend (optional). The package comprises several primary classes, including:

ClusterMapPlotter: This class enables the user to plot a basic heatmap and perform row and column clustering using different linkage methods and metrics. Additionally, users can add multiple annotations to the main heatmap's top, bottom, left, and right sides by providing a HeatmapAnnotation object as the parameter. Rows or columns can also be split according to metadata or hierarchical clustering.

HeatmapAnnotation: This class can be passed to [left/right/top/bottom]_annotation in ClusterMapPlotter or DotClustermapPlotter. HeatmapAnnotations allows users to add boxplot, barplot, and scatter plot annotations to the heatmap. Label annotations can also be added, and the anno_label merges the labels of the samples belonging to the same group and distributes them evenly throughout the axis without overlapping. Custom annotation classes can be added, and users can include as many kinds of annotations as desired.

col_ha = HeatmapAnnotation(…,axis=**1**, cmap='set1', label_kws = {…}, …)

row_ha = HeatmapAnnotation(…,axis=**0**, cmap='set1', label_kws = {…}, …)

The main heatmap can be accompanied by heatmap annotations, which can either be plotted alongside the main heatmap or separately. For instance, PyComplexHeatmap can be used to visualize categorical and continuous data simultaneously in Figure 1B by incorporating heatmap annotations. Anno_barplot can automatically determine whether to generate a bar plot or a stacked bar plot depending on the structure of the input data frame.

DotClustermapPlotter: A class inherited from ClusterMapPlotter to plot dot heatmap using a similar input style as seaborn. The user can visualize up to five‐dimensional biological data, including the *x‐* and *y*‐axis, the size, colormap, marker, and hue of the dot by providing a pandas data frame and other parameters.

oncoPrintPlotter: A class to plot oncoPrint plot, which was also inherited from ClusterMapPlotter. In addition to OncoPrint, users can also visualize any other categorical data using oncoPrintPlotter.

Composite: The composite function is used to combine two or more ClusterMapPlotter objects horizontally or vertically as a joint figure without any manual editing.

composite(cmlist = [cm1, cm2], main=**0**, ax=None, axis=**1**,…)

### Applications

The heatmap in Figure 1C displays annotations for both rows (anno_scatterplot, anno_barplot, and anno_label) and columns (anno_simple and anno_boxplot) using simulated datasets consisting of randomly generated normal distributed data. The samples were divided into two groups using the parameter col_split = df_col.AB, while rows were segregated into two groups based on the clustering tree structure generated by scipy.cluster.hierarchy.fcluster, using row_split = 2. On the right side of the heatmap, the scatter plot, and barplot annotations exhibit the data for sample 4, with labels displayed for selected features where sample 4 > 0.5.

Figure 1D illustrates the methylation signatures specific to different cell types. The CpG signatures and data were obtained from Loyfer et al. [16]. The label annotations on the top of this heatmap were added using anno_label as part of the column annotation. Labels from different samples belonging to the same cell group were merged and distributed throughout the *x*‐axis with a simple line of code.

anno_label(df_col.Group, merge=True, rotation=90, extend=True, colors=col_colors_dict, …)

**Figure 2:**
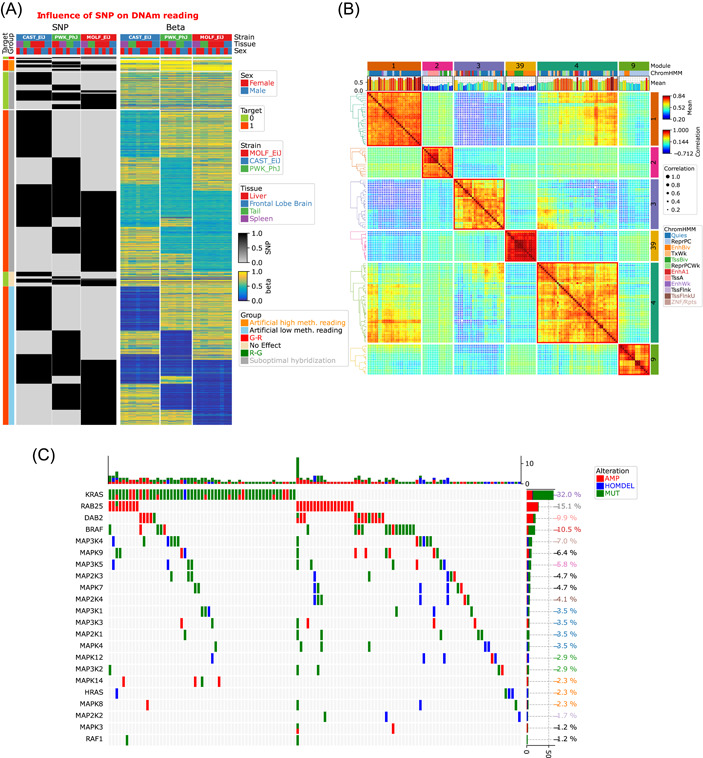
Illustration of heatmaps composition, dot heatmap, and oncoPrint. (A) The influence of mouse strain‐specific SNP on DNA methylation reading of MM285 array. Two heatmaps were combined into a joint figure. (B) Dot heatmap showing the correlation matrix for CpGs coming from different modules. (C) OncoPrint of the TCGA (The Cancer Genome Atlas) lung adenocarcinoma carcinoma variants profile.

In Figure 2A, we demonstrate the influence of strain‐specific SNP on mouse array DNA methylation reading [15]. To represent the three wild mouse strains, all samples were divided into three groups using strain as the column split criterion. Probes with SNP were further divided into multiple groups based on the combination of target and group as rows split. By setting add_text = True in the anno_simple, strain names can be added easily to the top of the simple annotation. The left panel illustrates the SNP pattern in different probes across various strains, while the right panel displays the actual DNA methylation beta values for the corresponding probes and samples. We generated two panels separately using ClusterMapPlotter and combined them using the composite function to form a joint figure.

Figure 2B utilized a dot cluster map (DotClustermapPlotter) to illustrate the correlation matrix of six CpG modules identified through the KnowYourCG package (https://www.bioconductor.org/packages/release/bioc/vignettes/sesame/inst/doc/KYCG.html). The size of the dots on the heatmap represented the correlation coefficient (*r*), and a variety of annotations were incorporated. Furthermore, the dot heatmap was divided into separate clusters based on the CpG modules. The different modules were enriched in distinct ChromHMM categories and exhibited varying mean DNA methylation levels, as demonstrated in the top annotation.

Using the PyComplexHeatmap layout as a foundation, we can create the oncoPrint heatmap, which is derived from the ClusterMapPlotter with some adjustments. Figure 2C illustrates the visualization of TCGA (The Cancer Genome Atlas) lung adenocarcinoma carcinoma variants profile through the oncoPrint heatmap. Like the DotClustermapPlotter and ClusterMapPlotter, various annotations can be added to the oncoPrint and the heatmap can also be split. Furthermore, it is possible to merge two or more oncoPrint heatmaps horizontally or vertically.

We developed this package as an initial step towards creating complex heatmaps in Python, and we believe that with the help of the open‐source community on Github, it can be enhanced further. For more examples, please refer to the documentation site: https://dingwb.github.io/PyComplexHeatmap.

## DISCUSSION

As single‐cell sequencing sample sizes continue to increase, more Python packages are emerging to aid in the processing and analysis of single‐cell genomic and epigenomic datasets. This manuscript introduces PyComplexHeatmap, a Python package that simplifies and accelerates the generation of complex heatmaps in Python. Our package was compared to state‐of‐the‐art data visualization packages, including matplotlib, seaborn, and ComplexHeatmap (Supporting Information: Table S1). PyComplexHeatmap currently boasts the most robust heatmap visualization capabilities in Python, with most of the features not available in other Python packages. In comparison to the R package ComplexHeatmap, many features in PyComplexHeatmap, such as automatically adding text above annotations, automatically distributing annotation labels, and generating dot heatmaps, are easier to use and can be produced automatically. When comparing running time and memory usage, PyComplexHeatmap performs better than R ComplexHeatmap when visualizing the same data set using the same clustering method and metric, running faster and consuming only one‐third of the memory (Table 1). The data set and code utilized to compare these two packages can be found at https://github.com/DingWB/PyComplexHeatmap/tree/main/comparison.

**Table 1:** Comparison of the processing time and maximal memory usage for PyComplexHeatmap and ComplexHeatmap.

Package name	Processing time (s)	Memory (kb)
ComplexHeatmap	40.21	3,366,768
PyComplexHeatmap	22.57	1,037,944

While PyComplexHeatmap has some advantages, it is not as versatile as R ComplexHeatmap. Our package provides a framework for developing various heatmap visualization functions, and we anticipate that further efforts will be required by us and the Python community to optimize and update the package, including supporting more flexible input data format, implementing parallel computing and plotting, integrating more clustering methods, accommodating polar axes, providing interactive visualization, and integrating with other Python packages such as Scanpy, Django in the future.

## CONCLUSION

We introduced PyComplexHeatmap, a versatile and user‐friendly Python package, to fill the multidimensional data visualization gap in the Python‐based data science ecosystem. We showcased the main features of PyComplexHeatmap in rendering complex biological datasets with rich annotations. Our benchmark suggested a superior computational efficiency over the R implementation. We demonstrated its power in advanced genomics data analysis, including rendering the OncoPrint plot for cancer genomics data analysis and dissecting single‐cell multiomics data of five dimensions.

## AUTHOR CONTRIBUTIONS

**Wubin Ding**: Algorithm implementation, writing: original draft preparation and reviewing. **David Goldberg**: Data preparation. **Wanding Zhou**: writing: reviewing and editing.

## CONFLICT OF INTEREST STATEMENT

The authors declare no conflict of interest.

## Supporting information

Supplementary Table S1: A table summarizes the features that are included in PyComplexHeatmap and compare it to other packages: Matplotlib, Seaborn, and ComplexHeatmap.

## Data Availability

Data sharing is not applicable to this article as no new data were created or analyzed in this study. The PyComplexHeatmap package and documentation are available on Github: https://github.com/DingWB/PyComplexHeatmap. Supplementary materials (figures, tables, scripts, graphical abstract, slides, videos, Chinese translated version, and updated materials) may be found in the online DOI or iMeta Science http://www.imeta.science/.
